# Facile Preparation of Cu_2_O Nanoparticles and Reduced Graphene Oxide Nanocomposite for Electrochemical Sensing of Rhodamine B

**DOI:** 10.3390/nano9070958

**Published:** 2019-06-30

**Authors:** Quanguo He, Jun Liu, Yaling Tian, Yiyong Wu, Felista Magesa, Peihong Deng, Guangli Li

**Affiliations:** 1 School of Life Science and Chemistry, Hunan University of Technology, Zhuzhou 412007, China; 2 Key Laboratory of Functional Metal‒Organic Compounds of Hunan Province; Key Laboratory of Functional Organometallic Materials of Hunan Provincial Universities; Department of Chemistry and Material Science, Hengyang Normal University, Hengyang 421008, China; 3 School of Life Sciences and Bioengineering, Nelson Mandela African Institution of Science and Technology, Arusha P.O.BOX 447, Tanzania

**Keywords:** electroanalysis, Cu_2_O nanoparticles, rhodamine B, reduced graphene oxide

## Abstract

In this paper, the preparation, characterization, and electrochemical application of Cu_2_O nanoparticles and an electrochemical reduced graphene oxide nanohybrid modified glassy carbon electrode (denoted as Cu_2_O NPs‒ERGO/GCE) are described. This modified electrode was used as an electrochemical sensor for the catalytic oxidation of rhodamine B (RhB), and it exhibited an excellent electrochemical performance for RhB. The oxidation potential of RhB was decreased greatly, and the sensitivity to detect RhB was improved significantly. Under optimum conditions, a linear dynamic range of 0.01–20.0 μM and a low detection limit of 0.006 μM were obtained with the Cu_2_O NPs‒ERGO/GCE by using second‒order derivative linear sweep voltammetry. In addition, the selectivity of the prepared modified electrode was analyzed for the determination of RhB. The practical application of this sensor was investigated for the determination of RhB in food samples, and satisfactory results were obtained.

## 1. Introduction

Rhodamine B (RhB), as a synthetic organic dye, is widely used in paper, textile, porcelain, leather, and paint industries [1]. However, studies have shown that RhB is carcinogenic. According to the International Agency for Research on Cancer (IARC), RhB carries a carcinogenic risk: The inhalation, ingestion, and skin contact of RhB may lead to acute and chronic poisoning as well as injury [2]. The concentration of RhB in industrial wastewater is generally higher than 100 mg/L and difficult to biodegrade [3], which not only causes damage to plants and animals but also poses a serious health threat to human. Europe, the United States, and China have made it clear that it is not allowed to be used in foodstuffs [4,5,6]. However, due to the low price, bright color, and strong stability of RhB, the illegal use of RhB has still been found in food in recent years, endangering the health of consumers. Therefore, the determination of RhB in wastewater and foodstuffs is very important for ensuring the safety of human beings.

At present, the analytical methods for RhB determination are mainly high performance liquid chromatography [7], electrophoresis [8], UV‒Vis spectrometry [9,10,11], surface–enhanced Raman scattering spectroscopy [12], and fluorescence spectroscopy [13]. Though the detection limits of most methods are low, complicated sample pretreatment methods makes experiments time‒consuming and not suitable for real‒time or field monitoring. In addition, complex and expensive instruments and skilled operators are necessary for the above methods. Electrochemical analysis has the advantage of rapid response, cheap instruments, simple operation, time‒savings, high sensitivity, and good selectivity. However, the direct detection of RhB using electrochemical methods is rare. Nowadays, only five modified glass carbon electrodes (GCEs) have been reported for the detection of RhB [14,15,16,17,18]. Though these modified electrodes can significantly improve the electrocatalytic performance of RhB, the electrode preparation is complex and has a high cost, both of which limit its practical applications and make it commercially unfavorable. Therefore, developing simple and environmental friendly routes to prepare novel modified electrodes for RhB detection is still a highly demanded challenge.

In recent years, metal oxide nanoparticles have attracted the extensive attention of many researchers due to their high catalytic performance, low cost, and good stability [19,20,21,22,23,24,25,26]. Cu_2_O is an important p‒type semiconductor. It has a narrow band gap and is easily excited by visible light. Cu_2_O has many advantages such as photochemical stability, non‒toxicity, and low cost, which allow it to have potential applications in new energy, photocatalytic degradation, sterilization, sensing, and other fields [27,28,29,30,31]. Ma et al. [32] successfully prepared Cu_2_O with a cubic shape and fabricated a Nafion/Cu_2_O/GCE by a dropping coating method. The developed sensor was successfully applied to detect glucose in real urine samples. Selvarajan et al. [33] prepared a silver/polypyrrole/cuprous oxide ternary nanocomposite (Ag/PPy/Cu_2_O) modified electrode which utilized the excellent catalytic performance of Cu_2_O to realize the sensitive and selective detection of serotonin. Jin et al. [34] prepared a modified electrode based on Cu_2_O@Pt core‒shell nanoparticles. The modified electrode showed good electrocatalytic activity for dopamine. The linear range was 10 nM–1027.16 µM, and the detection limit was 3 nM. However, the application of Cu_2_O nanoparticles (Cu_2_O NPs) in electrochemical sensors is seriously hindered because of their poor conductivity and uneven dispersion. In order to reduce their charge transfer resistance and improve their electrochemical performance, great efforts have recently been dedicated to combining conductive materials with Cu_2_O NPs.

Graphene (GR) is a new type of two‒dimensional atomic crystal which consists of a single layer of carbon atoms. Theoretically, the specific surface area of GR is up to 2630 m^2^/g, and the thickness is only 0.35 nm, which makes it an ideal template for the synthesis of GR matrix composites. It has been widely studied in battery, photocatalysis, sensing, and other fields [35,36,37]. Liu et al. [38] prepared Cu_2_O‒GR nanocomposites using hydroxylamine hydrochloride as a reducing agent, and these nanocomposites were applied in detecting glucose and hydrogen peroxide. Xu et al. [39] constructed a non‒enzymatic hydrogen peroxide sensor based on Cu_2_O‒GR nanocomposites using physical adsorption, in‒situ reduction, and one‒pot synthesis. Zhang et al. [40] successfully prepared Cu_2_O‒GR nanocomposites by a solvothermal method for the sensitive detection of dopamine. However, the above mentioned methods require more synthesis steps, and a one‒step green synthesis of Cu_2_O‒GR nanocomposites for RhB detection has not yet been reported.

In this work, graphene oxide (GO) was used as a carrier for Cu_2_O NPs. The surface of GO contains a large number of oxygen‒containing functional groups, including carboxyl and carbonyl groups, which can provide nucleation sites for loading Cu_2_O NPs. Therefore, Cu_2_O NPs can be firmly attached to the GO surface. Then, the Cu_2_O NPs‒electrochemical reduced graphene oxide (ERGO) nanocomposites were synthesized in one step by a potentiostatic reduction method, which has the advantages of being simple, green, and pollution‒free. The obtained material exhibited a good performance for the sensitive detection of RhB. In addition, the developed method has many advantages such as rapidness, low cost, high sensitivity, and good selectivity, and satisfactory results were achieved in the real samples analyses.

## 2. Experimental

### 2.1. Reagents

Graphite powder, polyvinylpyrrolidone, cupric sulfate pentahydrate (CuSO_4_·5H_2_O), and RhB were purchased from Sinopharm Chemical Reagent Company (Shanghai, China). 0.0479 g of RhB was accurately weighed and dissolved in water, which was diluted to 100.0 mL to prepare the standard solution of 1.0 mM. The low concentration working solutions of RhB were prepared by diluting the standard solution. A 0.15 M HAc–NaAc buffer (pH 6.6) was used as a supporting electrolyte. All chemicals were of analytical grade and did not need further purification when used. All the water used was doubly distilled water.

### 2.2. Instruments

Cyclic voltammetry (CV) was carried out on a CHI 660E electrochemical workstation of Shanghai Chenhua Instruments Company, China. The second derivative linear scan voltammetry was determined on a JP‒303E polarographic analyzer (Chengdu Instrument Factory, Chengdu, China). All electrochemical measurements were performed using a three‒electrode system (including a working electrode (Cu_2_O NPs‒ERGO/GCE), a reference electrode (saturated calomel electrode, SCE), and a counter electrode (platinum electrode). The pH value was measured on a pH‒3c exact digital pH meter (Leichi Instrument Factory, Shanghai, China). A scanning electron microscope (EVO10, ZEISS, Jena, Germany) was used to obtain scanning electron microscopy (SEM) images at 2.0 KV of acceleration voltage. A powder X‒ray diffractometer (PANalytical, Amsterdam, Holland) with Cu Kα radiation (0.1542 nm) was employed to analyze the crystal structure of Cu_2_O. High performance liquid chromatography (HPLC) analysis was carried out using an Agilent 1100 series HPLC system and fluorescence detector (Agilent Technology Co., Ltd., Beijing, China).

### 2.3. Preparation of Cu_2_O NPs‒GO Dispersion

GO was synthesized according to our previous report [41]. Cu_2_O NPs were synthesized according to Xu et al. [33]. Typically, 100 mg of CuSO_4_·5H_2_O and 50 mg of polyvinylpyrrolidone were dissolved in 20 mL of distilled water and stirred for 30 min, and then 4 mL of 0.2 M NaOH was added to the above solution slowly and stirred continuously for another 30 min to obtain a blue precipitate. Finally, 15 µL of hydrazine hydrate solution (80 wt%) was added to the mixture and stirred continuously for 20 min to obtain a brick red suspension. The suspension was centrifuged and washed with anhydrous ethanol and ultrapure water in turn, and then it was dried at room temperature. Finally, 1.0 mg of Cu_2_O‒NPs were dispersed in 20 mL of a GO solution (1 mg/mL), and the uniform Cu_2_O‒GO dispersion was obtained by ultrasonication for 2 h.

### 2.4. Electrode Preparation

The 3 mm in diameter glassy carbon electrode (GCE) was polished with 0.05 µm of alumina slurry and then washed with distilled water, absolute ethanol, distilled water in an ultrasonic bath—each for 1 min—and dried under an infrared lamp. A 5 µL dispersion of Cu_2_O NPs‒GO was dried on the surface of the GCE at room temperature. The Cu_2_O NPs‒ERGO/GCE was prepared by immersing the Cu_2_O NPs‒GO/GCE in a phosphate buffer solution (pH 6.0) and reducing it for 120 s at a constant potential of −1.2 V. For comparison, other modified electrodes such as a GO/GCE, ERGO/GCE, and Cu_2_O NPs‒GO/GCE were prepared in a similar way.

### 2.5. Electrochemical Measurement

One milliliter of a RhB standard solution with an appropriate concentration was transferred to a 10 mL electrochemical cell, and 1.5 mL of a 1.0 M HAc–NaAc buffer (pH 6.6) and 7.5 mL of H_2_O were added. Then, the Cu_2_O NPs‒GO/GCE, an SCE, and a platinum electrode were inserted into the electrochemical cell. the measuring parameters were adjusted, and cyclic voltammetry or second derivative linear scan voltammetry were recorded. The calibration curve was established by plotting the relationship between the measured current signal and the analyte concentration. The content of RhB in sample solutions was determined by the standard addition method.

## 3. Results and Discussion

### 3.1. SEM and XRD Analysis

**Figure 1:**
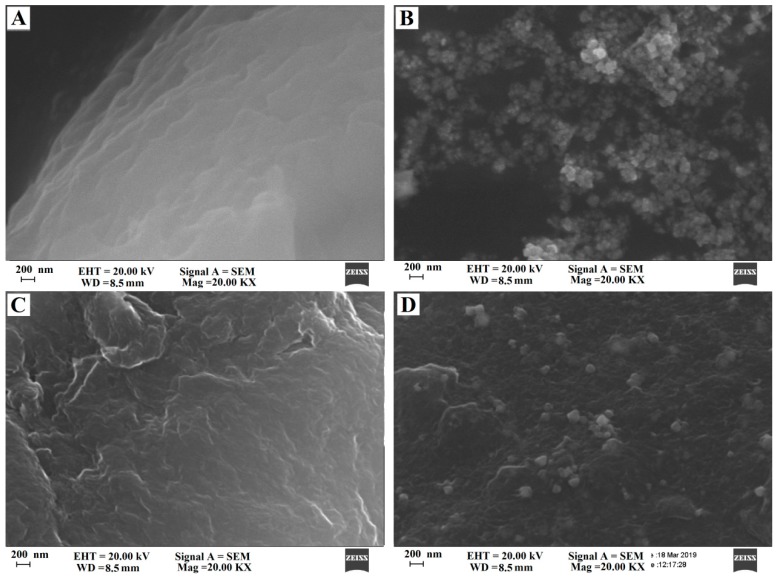
SEM images of (**A**) graphene oxide (GO), (**B**) Cu_2_O nanoparticles (NPs), (**C**) electrochemical reduced graphene oxide (ERGO), and (**D**) Cu_2_O NPs‒ERGO composites.

The SEM images of GO, Cu_2_O NPs, ERGO, and Cu_2_O NPs‒ERGO composites are illustrated in Figure 1. Figure 1A displays the image of the wrinkled multi‒layer graphene oxide, which corrugated and scrolled like crumpled silk veils. Figure 1B is the SEM image of pure Cu_2_O NPs. The product is spherical in shape with an average diameter of about 50–100 nanometers. Figure 1C shows the layered structure of ERGO nanosheets. The layered structure of ERGO could effectively increase the specific surface area of the modified electrode. It can be clearly seen from Figure 1D that the ERGO were decorated by Cu_2_O NPs. These spherical crystals were distributed randomly on the surface and edges of the ERGO sheets. In addition, the SEM images in Figure 1D show that the morphology and size of Cu_2_O NPs are similar to those observed in Figure 1B, which indicates that Cu_2_O NPs‒ERGO composites prepared by the electroreduction method would not change the structure of Cu_2_O.

**Figure 2:**
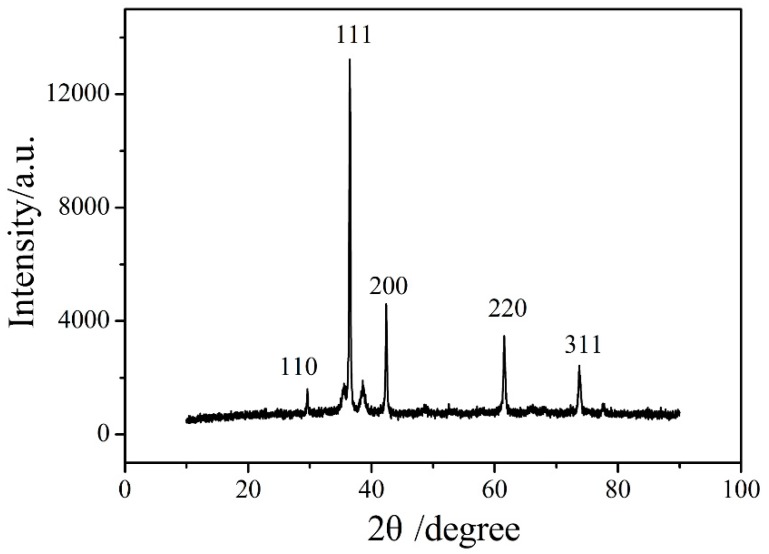
The X‒ray diffraction (XRD) pattern of Cu_2_O NPs.

The X‒ray Diffraction (XRD) pattern of Cu_2_O NPs is as shown in Figure 2. Comparing with the standard document of Cu_2_O (JCPDS No. 05‒0667) [42], the diffraction peaks of the prepared Cu_2_O are basically the same as that of the standard document. The main peaks are sharp, which proves that the crystallinity of Cu_2_O is relatively good. No diffraction peaks of other possible impurities (such as Cu and CuO) were detected, indicating that the product was pure Cu_2_O.

### 3.2. Characterization by CV

**Figure 3:**
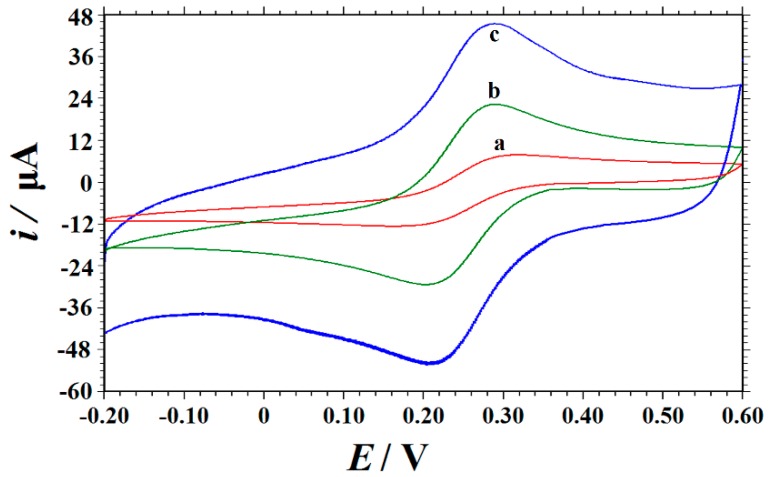
Cyclic voltammograms obtained on (**a**) a glassy carbon electrode (GCE), (**b**) ERGO/GCE, and (**c**) Cu_2_O‒ERGO/GCE in a mixture solution of 1.0 mM K_3_[Fe(CN)_6_] and 0.5 M KCl at the scan rate of 0.1 V s^−1^.

The cyclic voltammetric behaviors of 1.0 mM K_3_[Fe(CN)_6_] containing 0.5 M of KCl at different electrodes were studied at a potential scan rate of 100 mV s^−1^. As shown in Figure 3, on the bare GCE, a pair of redox peaks of [Fe(CN)_6_]^3−/4−^ was observed with a peak‒to‒peak separation (Δ*E*_p_) of 158 mV (curve a). While on an ERGO/GCE, both cathodic and anodic peak currents increased obviously, while the Δ*E*_p_ value decreased to 85 mV (curve b) due to the large surface area and excellent electrical conductivity of ERGO present on the electrode surface (curve b). On the Cu_2_O‒ERGO/GCE (curve c), the electrochemical behavior of the [Fe(CN)_6_]^3−/4−^ was dramatically improved with a cathodic peak potential (*E*_pc_) of 0.204 V and anodic peak potential (*E*_pa_) of 0.292 V. The peak‒to‒peak separation (Δ*E*_p_) was 88 mV (curve c), indicating that the presence of the high conductivity of ERGO together with the good catalytic activity of Cu_2_O on the GCE surface can further promote electron transfer and improve the performance of the sensor. According to the Randles–Sevcik equation [43]: *i*_pc_ = (2.69 × 10^5^) *n*^3/2^ *D*^1/2^ *v*^1/2^ *AC*, where *i*_pc_ is the reduction peak current (A), *n* is the electrontransfer number, *A* is the electroactive surface area (cm^2^), *D* is the diffusion coefficient of K_3_[Fe(CN)_6_] in the solution (7.6 × 10^‒6^ cm^2^ s^−1^ [44]), *C* is the concentration of K_3_[Fe(CN)_6_] (mol cm^−3^), and *v* is the scan rate (V s^−1^). By exploring the redox peak current with scan rate, the average electroactive areas of the GCE, ERGO/GCE, and Cu_2_O‒ERGO/GCE were calculated as 0.04711 cm^2^, 0.1142 cm^2^, and 0.1463 cm^2^, respectively. The results further indicate that the presence of ERGO and Cu_2_O NPs greatly improved the effective area of the electrode surface, which led to the great enhancement of the electrochemical response on the modified electrode.

### 3.3. Electrochemical Behaviors of RhB on Cu_2_O NPs‒ERGO/GCE

**Figure 4:**
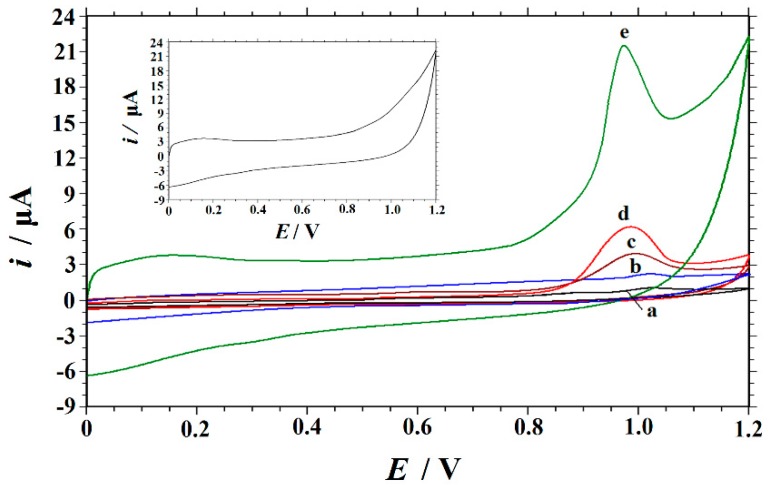
Cyclic voltammograms obtained with different modified electrodes in a 0.15 M HAc–NaAc buffer (pH 6.6) containing 10 μM of rhodamine B (RhB) at a scan rate of 0.1 V s^−1^. curve a: Bare GCE; curve b: GO/GCE; curve c: Cu_2_O NPs‒GO/GCE; curve d: ERGO/GCE; and curve e: Cu_2_O NPs‒ERGO/GCE. Inset: The cyclic voltammogram of Cu_2_O NPs‒ERGO/GCE in a 0.15 M HAc–NaAc buffer (pH 6.6).

The electrochemical behaviors of RhB at five different working electrodes in a 0.15 M HAc–NaAc buffer of pH 6.6 were compared by cyclic voltammetry, and the results are shown in Figure 4. As can be seen (in the inset in Figure 4), no redox peaks were observed on the Cu_2_O‒ERGO/GCE in a blank solution, indicating that the Cu_2_O‒ERGO/GCE is nonelectroactive in the selected potential region. On the other hand, when 10 μM of RhB was added into the blank solution, an oxidation peak was observed at each electrode in the scanning window of 0–1.2 V, indicating that RhB undergoes an irreversible redox process. On a bare GCE (curve a), RhB shows a poorly defined peak at 1.024 V with a very low current response (*i*_p_ = 0.6031 μA), which indicates a slow electron transfer. It can be seen that on the GO/GCE (curve b), the current response of RhB (*i*_p_ = 1.105 μA) was larger than that of the GCE, which may be due to the fact that the oxygen‒containing functional groups on GO nanosheets often act as catalytic active sites for some substances in the electrochemical reaction process [45,46]. On the Cu_2_O NPs‒GO/GCE (curve c), the oxidation peak current was bigger (*i*_p_ = 2.935 μA) than that of the GO/GCE, and the peak potential was negatively shifted to 0.995 V, indicating that Cu_2_O NPs could catalyze the electrochemical oxidation of RhB. An oxidation current peak of RhB at 0.986 V was obtained with the ERGO/GCE (curve d). The peak current (*i*_p_ = 5.338 μA) was enhanced by about nine times in comparison to that of the bare GCE. These findings indicate the excellent catalytic ability and good conductive performance of ERGO on the oxidation of RhB. With the Cu_2_O NPs‒ERGO/GCE (curve e), the charging current was obviously higher than that at the above electrodes, and the peak current of RhB was significantly higher (*i*_p_ = 13.884 μA). The peak current of RhB was enhanced by about 23 folds in comparison to that of the bare GCE. The peak current enhancement and the negative shift of the peak potential of RhB (0.970 V) were undoubtedly attributed to the characteristics of Cu_2_O NPs and ERGO. Specifically, the large specific surface area of ERGO increased the adsorption of RhB on the electrode surface. In addition, ERGO had good conductivity, which made up for the shortcomings of the Cu_2_O semiconductor. On the other hand, Cu_2_O loaded on the ERGO surface accelerated the electron exchange between RhB and the electrode surface, and it promoted the electrocatalytic reaction. Thus, these nanocomposites can be utilized to the maximum extent in the limited surface area of the electrode, providing an electron transfer microenvironment and realizing the sensitive determination of RhB.

### 3.4. The Effect of Potential Scan Rate

**Figure 5:**
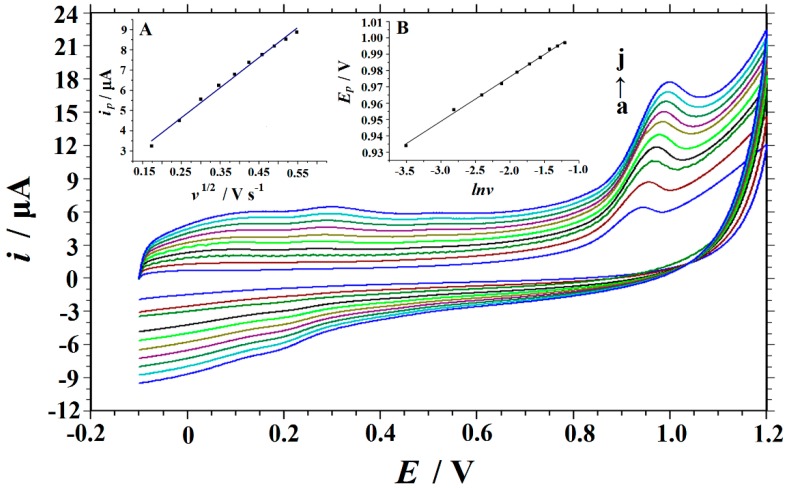
Cyclic voltammograms of 10 μM of RhB in a 0.15 M HAc–NaAc buffer (pH 6.6) obtained on the Cu_2_O NPs‒ERGO/GCE at different scan rates (**a**–**j**: 0.03, 0.06, 0.09, 0.12, 0.15, 0.18, 0.21, 0.24, 0.27, AND 0.3 V·s^−1^). Inset: (**A**) The plot of the peak current versus scan rate; (**B**) the plot of the peak potential versus the Napierian logarithm of scan rate.

The electrode reaction kinetics of RhB on the Cu_2_O NPs‒ERGO/GCE can be investigated by exploring the relationship between the scan rate and the electrochemical response. The cyclic voltammograms of 10 μM of RhB on the Cu_2_O NPs‒ERGO/GCE at different scan rates are shown in Figure 5. It can be seen form Figure 5 that when the scan rate increased from 0.03 V·s^−1^ to 0.3 V·s^−1^, the peak current increased gradually and had a linear relationship with the square root of the scan rate. The linear regression equation can be expressed as *i*_p_ = 14.851 *v*^1/2^ + 0.9307 (*i*_p_: μA, *v*: V s^−1^), and the correlation coefficient *R*^2^ was 0.9928, which indicates that the electrochemical process of RhB on the Cu_2_O NPs‒ERGO/GCE was controlled by diffusion. The diffusion control behavior was also confirmed by plotting log*i* vs. log*v*, and the corresponding linear equation is log*i* = 0.4338 log*v* + 1.185 (*R*^2^ = 0.996). The slope of 0.4338 was close to 0.5, which confirms the diffusion control characteristics of the electrode process. The electron transfer number of the electrode reaction can be calculated from the relationship between the peak potential and the scan rate [41]. As shown in the inset of Figure 5, the peak potential was linearly related to ln*v* in the range of 0.03–0.3 V·s^−1^. The linear regression equation was *E*_p_ = 0.0273 ln*v* + 1.0308 (Ep: V, v: V s^−1^), and the correlation coefficient R^2^ was 0.9977. For a totally irreversible diffusion‒controlled process, the slope of 0.0273 was equal to RT/2αnF [43], so αn = 0.47 can be calculated. α is generally considered to be 0.5 in a completely irreversible electrode process [46], so the number of electron transferred (n) that were involved in the oxidation process of RhB was about one.

### 3.5. Optimization of Determination Parameters

**Figure 6:**
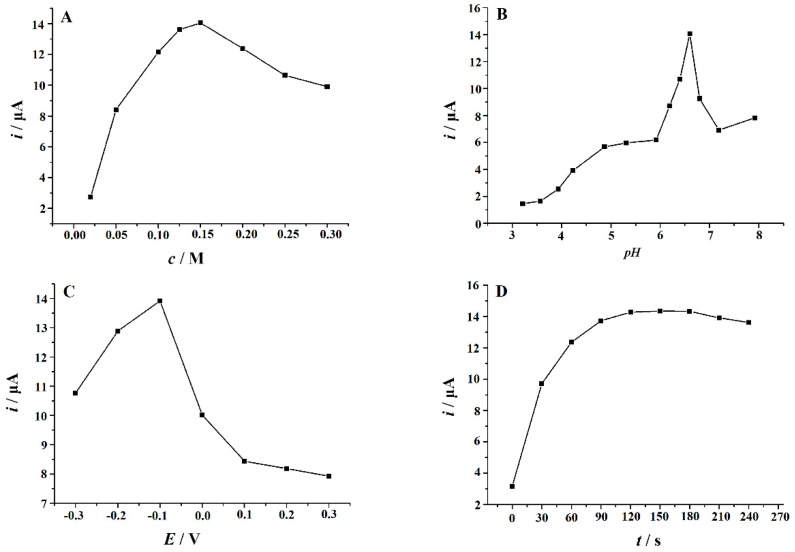
Effects of concentration of an HAc–NaAc buffer (**A**), solution pH (**B**), accumulation potential (**C**), and accumulation time (**D**) on the oxidation peak current of 10 μM of RhB on the Cu_2_O NPs‒ERGO/GCE. When one parameter changed, other parameters were at their optimal values.

In order to obtain the best determination conditions, the effects of various parameters on the electrochemical oxidation of RhB were studied. Firstly, the effects of the type and concentration of the supporting electrolyte were studied. The electrochemical responses of 10 μM of RhB were measured in different supporting electrolytes, which include an HAc–NaAc buffer (pH 3.0–8.0), an HAc–NH_4_Ac buffer (pH 3.0–8.0), a Britton–Robinson buffer (pH 3.0–8.0), a phosphate buffer (pH 3.0–8.0), NH_3_‒NH_4_Cl (pH 8.0–10.0), H_2_SO_4_, and HCl and HNO_3_ (each 0.1 M). It was found that the largest oxidation peak current and the best peak shape of RhB were obtained in an HAc–NaAc buffer. In addition, the effect of the concentration of the HAc–NaAc buffer on RhB oxidation was evaluated in the range of 0.02–0.6 M. It was found that with the increase of the concentration from 0.02–0.15 M, the peak oxidation current of RhB increased gradually, and then the current decreased when increasing the concentration from 0.15–0.6 M (Figure 6A). Therefore, 0.15 M was used as the best concentration of the HAc–NaAc buffer in this study. The effect of solution pH on the peak current of 10 μM of RhB was also investigated. The experimental results are shown in Figure 6B. It can be seen that when the pH value of the HAc–NaAc buffer increased from 3.21 to 6.60, the peak current of RhB continued to increase. However, when the pH value was higher than 6.60, the peak current began to decrease. Therefore, the optimal pH value of the HAc–NaAc buffer was 6.60. The effects of accumulation potential and time on 10 μM of RhB were also studied. Firstly, the accumulated potential was changed in the range from −0.3 to 0.3 V with a fixed accumulation time of 120 s. It was found that when the accumulated potential increased from −0.3 V to −0.1 V, the peak current increased, and then the peak current decreased dramatically with the further positive shift of the accumulation potential from −0.1 V to 0.3 V (Figure 6C). Therefore, the best accumulation potential was chosen as −0.1 V. The effect of accumulation time on the oxidation peak current of RhB was investigated at −0.1 V. It was found that the peak current of RhB increased with the increase of accumulation time. However, increasing accumulation time after 120 s did not lead to significant changes in peak current (Figure 6D). Therefore, considering the sensitivity and analysis speed, 120 s was chosen for quantitative analysis.

### 3.6. Interference Studies

**Figure 7:**
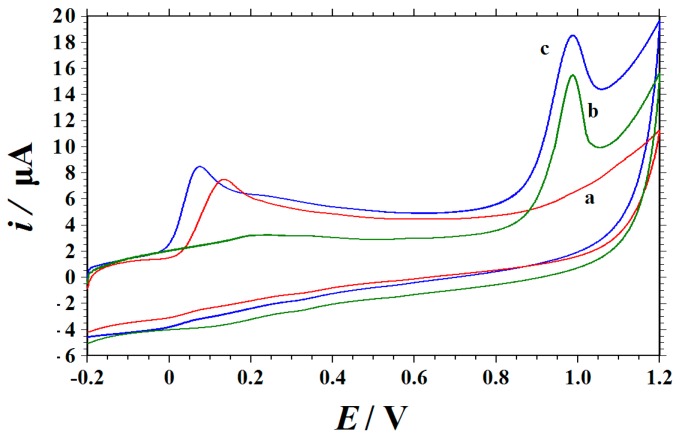
Cyclic voltammograms of 1.0 mM of AA (**a**), 10 μM of RhB (**b**), the mixed solution of 1.0 mM of AA and 10 μM of RhB on a Cu_2_O NPs‒ERGO/GCE in a 0.15 M HAc–NaAc buffer (pH 6.6) (**c**), with scan rate as 0.1 V s^−1^.

In order to evaluate the selectivity of the Cu_2_O NPs‒ERGO/GCE, the effect of some interfering substances on the electrochemical oxidation of RhB was studied. The response of the modified electrode to 10 μM of RhB in the presence of a 100‒fold concentration of Na^+^, Zn^2+^, K^+^, Mg^2+^, Ca^2+^, Cu^2+^, Cl^−^, SO_4_^2−^, glucose, sucrose, citric acid, and glycine were detected. The results showed that the above species did not cause significant interference (signal change ≤ ±5%). In addition, the interference of other dyes was tested. The experimental results indicated that a 100‒fold concentration of quinoline yellow; 10‒fold concentration of amaranth, ponceau 4R, allura red, sunset yellow, and lemon yellow did not interfere with the determination of 10 μM of RhB (signal change ≤ ±5%). Ascorbic acid(AA) is the main coexisting substance in food samples, so the electrochemical responses of RhB in the presence of AA on the Cu_2_O NPs‒ERGO/GCE were studied. The typical cyclic voltammogram of AA on a Cu_2_O NPs‒ERGO/GCE is shown in Figure 7 (curve a) and the oxidation peak appeared at 135 mV (vs. an SCE). Compared with the peak of RhB in Figure 7 (curve b), it can be seen that the oxidation peak was located at the different potential position (970 mV), and it did not interfere with the other peaks. Curve c was the cyclic voltammogram of AA and an RhB mixture solution on the Cu_2_O NPs‒ERGO/GCE, and two separated oxidation peaks appeared on the cyclic voltammogram with the potentials at 74 and 987 mV (vs. an SCE), which was attributed to that of AA and RhB, respectively (curve c). The oxidation peak potential separation was 913 mV for AA and RhB. These separations were large enough for simultaneous determinations of AA and RhB in the mixed solution. The above experimental data indicate that the proposed method has a good anti‒jamming ability.

### 3.7. Analytical Application

**Figure 8:**
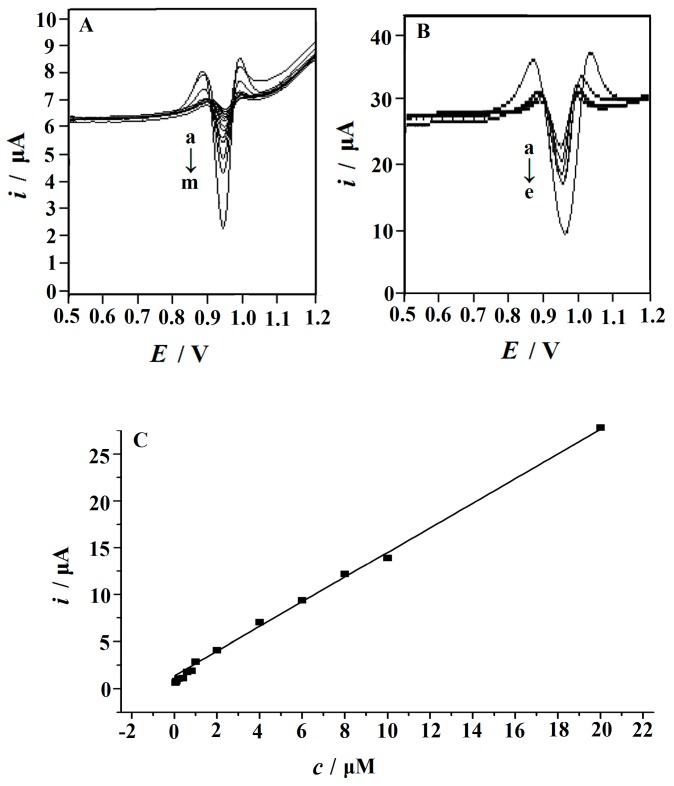
(**A**) Second‒order derivative linear scan voltammograms obtained with the Cu_2_O NPs‒ERGO/GCE in a 0.15 mol/L HAc–NaAc buffer (pH 6.6) containing different concentrations of RhB. From **a** to **e**: 4.0, 6.0, 8.0, 10, and 20 µM. The insets show the voltammograms at low concentrations (from **a** to **m**): 0.01, 0.02, 0.04, 0.06, 0.08, 0.1, 0.2, 0.4, 0.6, 0.8, 1.0, 2.0, and 4.0 µM. (**B**) The calibration plots of the concentration of RhB versus peak current. Accumulation potential: −0.1 V; accumulation time: 120 s, scan rate: 0.1 V s^−1^.

Under the optimum conditions, the second derivative linear sweep voltammetry was used to check the linear range and detection limit. Compared with square wave voltammetry (SWV) and differential pulse voltammetry (DPV), the main advantages of second derivative linear sweep voltammetry are a small background current and a sharp peak shape, both of which can significantly improve the sensitivity of determination and the overlapping resolution [47,48,49]. The second derivative linear sweep voltammograms of RhB at different concentrations are shown in Figure 8A. The linear range was between 0.01 and 20 μM. The linear regression equation can be expressed as *i*_p_ = 1.3511 *c* + 0.959 (*i*_p_: μA, c: μM), and the correlation coefficient R^2^ was 0.9979 (Figure 8B). Because of the large active surface area and strong accumulation ability, the detection limit of RhB was as low as 0.006 μM.

In order to compare with other electrochemical methods for RhB determination, Table 1 summarizes the performance of different modified electrodes. The results show that this method can provide a comparable linear range and detection limit with other modified electrodes. In addition, the Cu_2_O NPs‒ERGO/GCE described in our paper has the characteristics of fast response, simple electrode preparation, and good analytical performance.

The successive measurements of 10 μM of RhB were examined on a same Cu_2_O NPs‒ERGO/GCE. Unfortunately, the oxidation peak current of RhB continued to decrease, mainly due to the strong surface adsorption and fouling. However, the used Cu_2_O NPs‒ERGO/GCE could be recovered by cyclic scanning in a 0.1 M HNO_3_ solution in the range of 0–1.2 V for three to five cycles at a scanning rate of 0.1 V s^−1^. The relative standard deviation (RSD) was calculated to be 2.4% (*n* = 8). In addition, an RSD of 2.8% was obtained on 10 different Cu_2_O NPs‒ERGO/GCEs. These results showed that the method has good rrepeatability and reproducibility. In addition, the long‒term stability of the Cu_2_O NPs‒ERGO/GCE was tested. The experimental results showed that the current response of the modified electrode was 94.28% of the initial value after two weeks, which indicated that the modified electrode has high stability and can be used for RhB determination.

### 3.8. Detection of RhB in Real Samples

Using tomato juice, chili sauce, chili powder, and soy sauce as real samples, the practical application of the Cu_2_O NPs‒ERGO/GCE was evaluated. The samples were obtained from a local supermarket. The preparation of sample solutions was done according to reference [14]. A standard additional method was used to obtain the the ttypical results. In order to verify the accuracy of this method, the content of RhB was also analyzed by high performance liquid chromatography (HPLC). The results determined by HPLC and the Cu_2_O NPs‒ERGO/GCE are in good agreement (Table 2). In addition, a known amount of RhB was added to the sample solution, and a recovery test was carried out. The recoveries ranged from 96.3% to 103.0%, indicating that the Cu_2_O NPs‒ERGO/GCE has good analytical performance and can meet the requirements of RhB determination in food samples.

## 4. Conclusions

A sensitive and simple electrochemical method for the determination of RhB was developed using a Cu_2_O NPs‒ERGO/GCE. Based on the advantages of Cu_2_O NPs and ERGO, a Cu_2_O NPs‒ERGO/GCE has high electrocatalytic activity for the oxidation of RhB. Under optimum conditions, RhB was determined by second‒order derivative linear scan voltammograms with a wide linear range and a low detection limit. The method has good accuracy, acceptable precision, and reproducibility. This method provides a useful tool for on‒site monitoring of RhB in food samples.

## Figures and Tables

**Table 1:** Comparison of different modified electrodes for RhB.

Modified Electrode	Analytical Technique	Linear Range (µM)	Detection Limit (µM)	Reference
^a^ Cu@CS/GCE	^e^ DPV	0.3–30	0.1	[14]
^b^ MWCNTs‒COOH/IL/PGE	DPV	0.005–2.0; 2.0–60.0	0.001	[15]
^c^ SPZP/NAF/GCE	^f^ SWSV	0.01–5.0	0.0043	[16]
^d^ β‒CD‒AuNPs/HCNS/GCE	DPV	0.01–2	0.002	[17]
Cu_2_O NPs‒ERGO/GCE	Second derivative linear sweep voltammetry	0.01–20	0.006	This work

^a^ Cu@carbon sphere nanohybrid modified glassy carbon electrode; ^b^ carboxylated multi‒walled carbon nanotube and ionic liquid modified pencil‒graphite electrode; ^c^ silica‒pillared zirconium phosphate/nafion composite modified glassy carbon electrode; ^d^ per‒6‒thio‒β‒cyclodextrin functionalized nanogold/hollow carbon nanospheres nanohybrids modified glassy carbon electrode; ^e^ differential pulse voltammetry; ^f^ square‒wave stripping voltammetry.

**Table 2:** Determination of RhB in real food samples (*n* = 4).

Sample ^a^	Found by This Method ^b^/µM	Added/µM	Total Found by This Method ^b^/µM	Recovery/%	Determined by HPLC ^b^/µM
tomato juice	ND ^c^	5.0	5.08 (±0.19)	101.6	ND
chili sauce	2.67 (±0.09)	3.0	5.56 (±0.21)	96.3	2.64 (±0.10)
chili powder	0.81 (±0.02)	1.0	1.84 (±0.06)	103.0	0.85 (±0.03)
soy sauce	0.24 (±0.01)	0.2	0.442 (±0.02)	101.0	0.26 (±0.01)

**^a^** All samples were purchased from a local supermarket. **^b^** Average ± confidence interval, the confidence level is 95%. ^c^ Not detected.
